# Parity, breastfeeding, and breast cancer risk by hormone receptor status and molecular phenotype: results from the Nurses’ Health Studies

**DOI:** 10.1186/s13058-019-1119-y

**Published:** 2019-03-12

**Authors:** Renée T. Fortner, Julia Sisti, Boyang Chai, Laura C. Collins, Bernard Rosner, Susan E. Hankinson, Rulla M. Tamimi, A. Heather Eliassen

**Affiliations:** 1 Division of Cancer Epidemiology, German Cancer Research Center, Im Neuenheimer Feld 280, 69120 Heidelberg, Germany; 2 Channing Division of Network Medicine, Brigham and Women’s Hospital and Harvard Medical School, 181 Longwood Ave, Boston, MA 02115 USA; 3 Department of Epidemiology, Harvard T.H. Chan School of Public Health, 677 Huntington Ave, Boston, MA 02115 USA; 4 Department of Pathology, Beth Israel Deaconess Medical Center and Harvard Medical School, 330 Brookline Ave., Boston, MA 02215 USA; 5 Department of Biostatistics, Harvard T.H. Chan School of Public Health, 677 Huntington Ave, Boston, MA 02115 USA; 6 Department of Biostatistics and Epidemiology, School of Public Health and Health Sciences, University of Massachusetts Amherst, 715 North Pleasant St., Amherst, MA 01003 USA

**Keywords:** Breast cancer, Breastfeeding, Parity, Risk, Prospective cohort

## Abstract

**Background:**

Epidemiologic data suggest that parity increases risk of hormone receptor-negative breast cancer and that breastfeeding attenuates this association. Prospective data, particularly on the joint effects of higher parity and breastfeeding, are limited.

**Methods:**

We investigated parity, breastfeeding, and breast cancer risk by hormone-receptor (estrogen (ER) and progesterone receptor (PR)) and molecular subtypes (luminal A, luminal B, HER2-enriched, and basal-like) in the Nurses’ Health Study (NHS; 1976–2012) and NHSII (1989–2013). A total of 12,452 (ER+ *n* = 8235; ER− *n* = 1978) breast cancers were diagnosed among 199,514 women. We used Cox proportional hazards models, adjusted for breast cancer risk factors, to calculate hazard ratios (HR) and 95% confidence intervals (CI).

**Results:**

Parous women had lower risk of ER+ breast cancer (vs. nulliparous, HR = 0.82 [0.77–0.88]); no association was observed for ER− disease (0.98 [0.84–1.13]; *P*_het_ = 0.03). Among parous women, breastfeeding was associated with lower risk of ER− (vs. never 0.82 [0.74–0.91]), but not ER+, disease (0.99 [0.94–1.05]; *P*_het_ < 0.001). Compared to nulliparous women, higher parity was inversely associated with luminal B breast cancer regardless of breastfeeding (≥ 3 children: ever breastfed, 0.78 [0.62–0.98]; never breastfed, 0.76 [0.58–1.00]) and luminal A disease only among women who had breastfed (≥ 3 children, 0.84 [0.71–0.99]). Basal-like breast cancer risk was suggestively higher among women with higher parity who never breastfed; associations were null among those who ever breastfed.

**Conclusions:**

This study provides evidence that breastfeeding is inversely associated with hormone receptor-negative breast cancers, representing an accessible and cost-effective risk-reduction strategy for aggressive disease subtypes.

**Electronic supplementary material:**

The online version of this article (10.1186/s13058-019-1119-y) contains supplementary material, which is available to authorized users.

## Background

Data from recent prospective [1, 2], case-control [3–6], and case-only studies [7–9] suggest that parity increases risk of hormone receptor-negative breast cancer, while breastfeeding decreases risk (reviewed in [10]) or attenuates the detrimental effect of parity [3, 5, 11]. A general limitation of many prior prospective studies on these associations [1, 2, 12–16] is the inclusion of relatively few estrogen receptor (ER)-negative cases, given that only ~ 20% of tumors lack ER expression. Prospective data on the combination of higher parity and history of breastfeeding and ER− breast cancer risk are sparse [1, 11]. The African American Breast Cancer Epidemiology and Risk (AMBER) Consortium [11], including both case-control [4, 5] and cohort studies [1, 17], is the largest study to date (ER+, *n* = 2446; ER−, *n* = 1252). Within the consortium, parity was associated with increased risk of ER− breast cancer; this risk was attenuated among women who reported ever breastfeeding. Further, breastfeeding was only inversely associated with ER− disease; no association was observed for ER+ breast cancers.

To our knowledge, there are no prospective data on the combined effects of parity and breastfeeding by molecular phenotype (e.g., luminal subtypes, HER2-enriched, basal-like). Case-control [5, 7, 18] and case-only [9] studies suggest an increased risk of basal-like breast cancer only among parous women who did not breastfeed and an inverse association between breastfeeding and basal-like disease. In an earlier investigation of reproductive factors in the Nurses’ Health Studies (NHS, NHSII), we observed an inverse association between breastfeeding and basal-like breast cancer, but did not investigate the combined effect of parity and breastfeeding [13].

If breastfeeding is confirmed to reduce risk of hormone receptor-negative breast cancer, it represents an accessible and cost-effective risk-reduction strategy for this aggressive subtype. We hypothesized heterogeneous associations between parity and breastfeeding and ER+ and ER− breast cancer risk, with inverse associations hypothesized between parity and ER+ disease risk and between breastfeeding and ER− disease risk, and a positive association between parity and ER− breast cancer subtypes among women who did not breastfeed. Given intriguing, but inconsistent, prior results on the association between parity, breastfeeding, and breast cancer by subtype, and sparse prospective data on the combined effect of higher parity and breastfeeding, we examined these associations in the NHS and NHSII among almost 200,000 women.

## Methods

### Study population: the Nurses’ Health Study and Nurses’ Health Study II

The NHS was initiated in 1976 when 121,701 registered nurses, ages 30–55, completed and returned a mailed questionnaire [19]. The NHSII began in 1989 with 116,429 female registered nurses ages 25–42, using the same protocols. Participants have been followed biennially to update information on lifestyle and reproductive factors, and ascertain disease diagnoses. Cumulative follow-up rates are ≥ 94% in both cohorts.

### Parity and breastfeeding assessment

Participants reported pregnancies and breastfeeding on the biennial questionnaire (pregnancies: NHS 1976–1984, 1996, and NHSII 1989–2009; breastfeeding NHS 1986 and NHSII 1993, 1997). Parity was investigated as nulliparous vs. parous and total number of pregnancies (1, 2, 3, 4+ children); breastfeeding was investigated as nulliparous/never vs. ever and total duration (< 6, 7–11, ≥ 12 months). Further, we cross-classified parity with breastfeeding (nulliparous, parous/ever breastfed, parous/never breastfed; and nulliparous, never breastfed and 1, 2, or ≥ 3 children, ever breastfed and 1, 2, or ≥ 3 children).

### Covariate assessment

Age at each questionnaire was calculated using date of birth and questionnaire return date. Age at menarche and height were collected on baseline questionnaires. Other covariates included (date of collection) weight at age 18 (NHS 1980; NHSII 1989), height (NHS 1976; NHSII 1989), weight (biennially), oral contraceptive use (OC; NHS biennially until 1982; NHSII biennially), postmenopausal hormone therapy (HT; biennially), alcohol consumption (every 4 years, beginning in NHS 1980; NHSII 1991), age at first birth (NHS 1976 and biennially until 1982; NHS 1989 and biennially until 2011), menopausal status and age at menopause (biennially), diagnosis of benign breast disease (biennially), and family history of breast cancer (NHS 1982, and every 4 years beginning in 1988; NHSII 1989, and every 4 years beginning in 1997). Body mass index (BMI, kg/m^2^) at age 18 was calculated using self-reported weight and height.

### Breast cancer case ascertainment

Participants reported disease status on the biennial questionnaires. Cases included in this analysis reported no prior cancer diagnosis (except non-melanoma skin) and were diagnosed with invasive breast cancer through June 2014 (NHS) or June 2013 (NHSII). A total of 12,452 (NHS *n* = 8807; NHSII *n* = 3645) incident breast cancer cases were identified; 82% had data on tumor ER status (ER+ *n* = 8235; ER− *n* = 1978). A study physician confirmed breast cancer cases and extracted invasive vs. in situ, hormone receptor status, and tumor characteristics from the medical record. Vital status was ascertained through June 2015, using the National Death Index. Of the 12,452 breast cancer cases identified, 11,369 (91%) were confirmed through medical record review; remaining cases were participant confirmed. Given the high confirmation rate by medical record for breast cancer in this cohort (99%), both medical record and participant-confirmed cases are included.

### Tumor tissue collection

Details of tumor tissue collection have been detailed previously [20]. Tumor tissue samples were available from 4058 breast cancer cases (NHS, *n* = 3126; NHSII, *n* = 932). Tissue microarrays (TMAs) were created at the Dana Farber/Harvard Cancer Center (Boston, MA) with three 0.6-mm cores taken from each breast tumor included in the TMA block.

### Immunohistochemical (IHC) analyses

IHC staining for ER, progesterone receptor (PR), human epidermal growth factor receptor 2 (HER2), epidermal growth factor receptor (EGFR), and cytokeratin (CK) 5/6 was performed using a Dako Autostainer (Dako Corporation, Carpinteria, CA). IHC methods have been described previously [21]. Immunostained TMA slides were evaluated by a study pathologist. Tumor sections with any nuclear staining for ER or PR were classified as ER+ or PR+; ER− or PR− had complete absence of staining. Tumors were considered HER2+ when more than 10% of cells showed moderate or strong staining. Tumors were classified as CK5/6+ or EGFR+ if there was evidence of cytoplasmic or membranous staining.

### Classification of molecular subtypes

ER, PR, HER2, CK5/6, EGFR, and tumor grade were used to classify tumors into molecular subtypes; classification by IHC is a reasonable proxy for gene expression data [22–24]. Cases that were ER+ and/or PR+, HER2− and of histologic grade 1 and 2 were classified as luminal A cancers; cases that were either (a) ER+ and/or PR+ and HER2+ or (b) ER+ and/or PR+, HER2−, and of histologic grade 3 were classified as luminal B; cases that were ER−, PR−, and HER2+ were classified as HER2-enriched; and cases that were negative for ER, PR, and HER2 and positive for CK 5/6 and/or EGFR were categorized as basal-like. Cases that lacked expression of all five markers were considered “unclassified.” Of the invasive tumors on tissue microarrays, 3454 could be classified into the luminal A (*n* = 1903; 55%), luminal B (*n* = 934; 27%), HER2-enriched (*n* = 186; 5%), basal-like (*n* = 341; 10%), or unclassified (*n* = 90; 3%) subtypes. The few unclassified tumors were excluded from further analyses.

### Statistical analysis

After exclusions for prior cancer (*n* = 4528) or missing data on parity, age at first birth, or breastfeeding (*n* = 34,088), 199,514 women remained in the analysis. We calculated person-years beginning at date of baseline questionnaire return and ending at the earliest of date of diagnosis of any cancer (except non-melanoma skin cancer), date of death, or end of follow-up (NHS: June 1, 2012; NHSII: June 1, 2013). We used multivariable Cox proportional hazards models to calculate hazard ratios (HR) and 95% confidence intervals (CI) for risk of breast cancer by hormone receptor subtype (ER+, ER−; ER+/PR+, ER+/PR−, ER−/PR−) and by molecular phenotype (luminal A, luminal B, HER2-enriched, basal-like). Models were stratified by age in months, follow-up year, and cohort. Covariates were updated whenever possible throughout follow-up. Covariates in the final models were height, BMI at age 18, weight change since age 18, age at menarche, age at natural menopause, menopausal status, HT use, alcohol consumption, total physical activity, family history of breast cancer, and history of benign breast disease. Models restricted to parous women were additionally adjusted for age at first birth; in sensitivity analyses, we adjusted for years between menarche and first birth and years between last birth and minimum of current age and age at menopause. In analyses restricted to parous women, parity and breastfeeding were mutually adjusted. We further evaluated associations for ER+ and ER− disease in subgroups defined by time between last pregnancy and diagnosis.

Heterogeneity (*P*_het_) was assessed using a likelihood ratio test comparing models assuming the same association between the exposures and breast cancer subtypes to one assuming different associations for disease subtypes [25].

*P* values were considered statistically significant at < 0.05; all statistical tests were two-sided. Analyses were conducted in SAS 9.3 (Cary, NC).

## Results

A total of 93% of NHS participants and 67% of NHSII participants were parous at study baseline; among parous women, the majority reported ever breastfeeding (63% NHS, 79% NHSII). Median ages at recruitment for nulliparous women were 43.1 years in the NHS and 32.6 years in the NHSII; parous women were somewhat older (e.g., 2+ children, ever breastfed, median age 42.8 years in the NHS and 35.5 in the NHSII) (Table 1).

**Table 1:** Characteristics of the Nurses’ Health Study (NHS) and NHSII participants at baseline (NHS: 1976; NHSII: 1989)

	Nulliparous	1 child never breastfed	1 child breastfed	2+ children never breastfed	2+ children breastfed
Nurses’ Health Study	(*n* = 7346)	(*n* = 3995)	(*n* = 3596)	(*n* = 29,702)	(*n* = 53,977)
Age, years^*^	43.1 (7.8)	42.6 (7.8)	41.0 (8.1)	43.1 (6.9)	42.8 (7.1)
Height, meters	1.6 (0.1)	1.6 (0.1)	1.6 (0.1)	1.6 (0.1)	1.6 (0.1)
Body mass index at age 18, kg/m^2^	21.5 (3.5)	21.5 (3.5)	21.4 (3.2)	21.4 (3.0)	21.4 (2.9)
Weight change since age 18, kg	5.7 (9.2)	6.0 (9.8)	5.9 (9.3)	6.2 (9.0)	6.3 (8.9)
Age at menarche, years	12.5 (1.5)	12.5 (1.5)	12.5 (1.5)	12.5 (1.4)	12.6 (1.4)
Parity, %	0	1	1	3.1 (1.3)	3.4 (1.5)
Breastfeeding duration among parous women, %					
Never breastfed		100	0	100	0
6 months or less		0	78	0	54
7–12 months		0	13	0	15
> 12 months		0	9	0	31
Age at first birth, years		28.2 (4.9)	28.5 (5.0)	24.9 (3.0)	24.8 (3.0)
Age at last birth, years		28.2 (4.9)	28.5 (5.0)	30.8 (4.3)	31.3 (4.3)
Postmenopausal, %	27	23	23	19	18
Age at menopause, years^†^	42.9 (8.2)	44.1 (7.0)	44.5 (7.2)	46.0 (5.9)	46.5 (5.7)
Alcohol, g/day	7.8 (12.0)	6.1 (10.4)	6.6 (11.4)	6.1 (10.3)	6.3 (10.4)
Total physical activity, MET-hours/week	14.5 (19.9)	13.4 (19.6)	13.8 (19.0)	13.2 (19.8)	14.5 (21.8)
History of benign breast disease, %	25	22	23	18	18
Family history of breast cancer, %	6	6	6	6	6
Nurses’ Health Study II	(*n* = 33,284)	(*n* = 4790)	(*n* = 11,471)	(*n* = 9365)	(*n* = 41,988)
Age, years^*^	32.6 (4.8)	34.8 (4.9)	33.4 (4.6)	36.8 (4.2)	35.5 (4.0)
Height, meters	1.7 (0.1)	1.6 (0.1)	1.6 (0.1)	1.6 (0.1)	1.6 (0.1)
Body mass index at age 18, kg/m^2^	21.8 (4.1)	21.5 (3.6)	21.0 (3.0)	21.3 (3.3)	21.0 (2.8)
Weight change since age 18, kg	6.6 (11.5)	8.6 (11.3)	7.6 (10.1)	9.2 (11.3)	8.0 (9.9)
Age at menarche, years	12.4 (1.5)	12.3 (1.4)	12.4 (1.4)	12.3 (1.4)	12.4 (1.4)
Parity, %	0	1	1	2.3 (0.6)	2.5 (0.7)
Breastfeeding duration among parous women, %					
Never breastfed		100	0	100	0
6 months or less		0	42	0	18
7–12 months		0	37	0	18
> 12 months		0	21	0	64
Age at first birth, years		25.8 (4.8)	28.4 (4.3)	23.7 (3.6)	24.9 (3.5)
Age at last birth, years		25.8 (4.8)	28.5 (4.3)	27.6 (3.8)	29.2 (3.7)
Postmenopausal, %	3	3	2	3	2
Age at menopause, years^†^	37.5 (5.5)	38.0 (3.6)	38.7 (3.7)	37.7 (3.1)	37.8 (3.7)
Alcohol, g/day	4.0 (7.0)	2.8 (5.7)	2.9 (5.3)	2.4 (5.2)	2.3 (4.7)
Total physical activity, MET-hours/week	29.7 (42.1)	23.3 (34.2)	23.4 (34.8)	22.0 (33.5)	21.6 (30.8)
History of benign breast disease, %	9	9	8	8	7
Family history of breast cancer, %	6	6	6	5	6

Values are means (SD) or percentages and are standardized to the age distribution of the study population. Values of polytomous variables may not sum to 100% due to rounding

*Value is not age adjusted

^†^Age at natural menopause or bilateral oophorectomy

The associations between parity, breastfeeding, and breast cancer risk differed by tumor ER status (*P*_het_ = 0.03). Parity was associated with lower risk of ER+ breast cancer (parous vs. nulliparous: HR = 0.82 [95% CI 0.77–0.88]) (Table 2), but not associated with ER− disease (HR = 0.98 [0.84–1.13]). In contrast, ever breastfeeding was only associated with reduced risk of ER− breast cancer (HR = 0.83 [0.75–0.92]), and not ER+ disease (HR = 0.99 [0.94–1.04]), relative to parous women who never breastfed and nulliparous women. Results were similar regardless of duration of breastfeeding (e.g., ≥ 12 months vs. never, ER− HR = 0.83 [0.73–0.94], *P*_trend_ = 0.05; ER+ HR = 1.02 [0.96–1.09], *P*_trend_ = 0.22). The association between parity and ER+ tumors did not differ by breastfeeding history (e.g., parous, never breastfed: HR = 0.83 [0.77–0.90]; parous, ever breastfed: HR = 0.82 [0.76–0.88]), and parity was not associated with ER− breast cancer risk in either breastfeeding history subgroup (parous, never breastfed: HR = 1.11 [0.94–1.31]; parous, ever breastfed: HR = 0.92 [0.79–1.08]).

**Table 2:** Multivariable-adjusted^a^ hazard ratios (HRs) and 95% confidence intervals for breast cancer overall and by ER status in relation to parity and breastfeeding: Nurses’ Health Study (1976–2012) and Nurses’ Health Study II (1989–2013)

	All			ER^+^			ER^−^			*P*_het_ by
	Cases	PY	HR (95% CI)	Cases	PY	HR (95% CI)	Cases	PY	HR (95% CI)	subtype
Parous										
Nulliparous	1498	742,502	1 (ref.)	979	742,976	1 (ref.)	209	743,672	1 (ref.)	
Parous	10,954	4,540,107	0.84 (0.80–0.89)	7256	4,543,555	0.82 (0.77–0.88)	1769	4,548,669	0.98 (0.84–1.13)	0.03
Breastfeeding										
Never breastfed (parous + nulliparous)	5101	2,100,966	1 (ref.)	3271	2,102,676	1 (ref.)	835	2,104,928	1 (ref.)	
Ever breastfed	7351	3,181,643	0.94 (0.91–0.98)	4964	3,183,855	0.99 (0.94–1.04)	1143	3,187,412	0.83 (0.75–0.92)	0.002
≤ 6 months	3242	1,255,908	0.93 (0.88–0.97)	2177	1,256,886	0.96 (0.90–1.01)	517	1,258,482	0.84 (0.75–0.95)	
7–11 months	1203	535,074	0.96 (0.90–1.03)	819	535,428	1.03 (0.95–1.11)	180	535,998	0.81 (0.68–0.95)	
≥ 12 months	2906	1,390,661	0.96 (0.91–1.01)	1968	1,391,541	1.02 (0.96–1.09)	446	1,392,932	0.83 (0.73–0.94)	
			*P*_trend_ = 0.72			*P*_trend_ = 0.22			*P*_trend_ = 0.05	0.02
Parity/breastfeeding										
Nulliparous	1498	742,502	1 (ref.)	979	742,976	1 (ref.)	209	743,672	1 (ref.)	
Parous women, never breastfed	3603	1,358,464	0.88 (0.83–0.94)	2292	1,359,700	0.83 (0.77–0.90)	626	1,361,257	1.11 (0.94–1.31)	< 0.001^b^
1 child	518	210,088	0.92 (0.83–1.02)	332	210,263	0.88 (0.78–1.00)	91	210,483	1.15 (0.90–1.48)	0.002^c^
2 children	1386	516,819	0.93 (0.86–1.00)	870	517,287	0.86 (0.78–0.94)	250	517,868	1.20 (0.99–1.45)	
≥ 3 children	1699	631,558	0.81 (0.76–0.87)	1090	632,150	0.77 (0.70–0.84)	285	632,906	1.03 (0.85–1.25)	
Parous women, ever breastfed	7351	3,181,643	0.83 (0.78–0.88)	4964	3,183,855	0.82 (0.76–0.88)	1143	3,187,412	0.92 (0.79–1.08)	
1 child	666	313,938	0.95 (0.86–1.04)	437	314,149	0.93 (0.83–1.04)	97	314,457	0.97 (0.76–1.23)	
2 children	2493	1,166,539	0.85 (0.79–0.91)	1694	1,167,283	0.85 (0.78–0.92)	362	1,168,492	0.87 (0.73–1.03)	
≥3 children	4192	1,701,166	0.79 (0.74–0.84)	2833	1,702,423	0.78 (0.72–0.84)	684	1,704,463	0.95 (0.81–1.12)	
Among parous women										
Parity^d^										
1 child	1184	524,026	1 (ref.)	769	524,412	1 (ref.)	188	524,940	1 (ref.)	
2 children	3879	1,683,357	1.02 (0.95–1.09)	2564	1,684,570	1.02 (0.94–1.11)	612	1,686,360	1.02 (0.86–1.21)	
3 children	3165	1,259,779	1.03 (0.96–1.10)	2120	1,260,791	1.03 (0.94–1.13)	502	1,262,311	1.06 (0.88–1.27)	
≥ 4 children	2726	1,072,945	0.92 (0.85–1.00)	1803	1,073,782	0.91 (0.82–1.00)	467	1,075,059	1.08 (0.89–1.31)	
			*P*_trend_ = 0.0001			*P*_trend_ = 0.0001			*P*_trend_ = 0.43	0.02
Breastfeeding^d^										
Never breastfed	3603	1,358,464	1 (ref.)	2292	1,359,700	1 (ref.)	626	1,361,257	1 (ref.)	
Ever breastfed	7351	3,181,643	0.95 (0.91–0.99)	4964	3,183,855	0.99 (0.94–1.05)	1143	3,187,412	0.82 (0.74–0.91)	< 0.001
≤ 6 months	3242	1,255,908	0.93 (0.89–0.98)	2177	1,256,886	0.96 (0.91–1.02)	517	1,258,482	0.84 (0.75–0.95)	
7–11 months	1203	535,074	0.95 (0.89–1.02)	819	535,428	1.02 (0.94–1.10)	180	535,998	0.79 (0.67–0.94)	
≥ 12 months	2906	1,390,661	0.97 (0.92–1.02)	1968	1,391,541	1.04 (0.97–1.11)	446	1,392,932	0.80 (0.70–0.92)	
			*P*_trend_, incl never = 0.70			*P*_trend_, incl never = 0.05(+)			*P*_trend_, incl never = 0.02	0.002
			*P*_trend_, among ever = 0.10			*P*_trend_, among ever = 0.02(+)			*P*_trend_, among ever = 0.35	

^a^Adjusted for age, height (< 1.60, 1.60 to < 1.65, 1.65 to < 1.70, 1.70 to < 1.75, 1.75+ meters), BMI at age 18 (< 20, 20–21.9, 22–23.9, 24–26.9, 27+ kg/m^2^), weight change since age 18 (continuous, kg), age at menarche (< 12, 12, 13, 14, 15+ years), menopausal status (premenopausal, postmenopausal or unknown), age at natural menopause (continuous), HT use (never, past, current E only, current E + P, current other), alcohol consumption (non-drinker, < 5, 5–10, 10–15, 15+ g/day), total physical activity (< 3, 3–9, 9–18, 18–27, 27+ MET-h/day), family history of breast cancer (yes/no), history of benign breast disease (yes/no)

^b^*P*_het_ for nulliparous and parous ever vs. never breastfed

^c^*P*_het_ for nulliparous and number of children by ever vs. never breastfed

^d^Among parous women only, additionally adjusted for age at first birth (continuous, years), and parity and breastfeeding mutually adjusted

We next restricted the analyses to parous women and evaluated number of children, breastfeeding, and breast cancer risk (Table 2). Relative to women with parity of 1, only parity of 4 or higher was associated with lower ER+ breast cancer risk (≥ 4 children HR = 0.91 [0.82–1.00]). No association was observed in the other parity categories (e.g., 3 children HR = 1.03 [0.94–1.13]), or in any category for ER− breast cancer. The association between breastfeeding and breast cancer risk among parous women was similar to that observed in the analysis among all participants, including nulliparous women (e.g., ever vs. never, ER+ HR = 0.99 [0.94–1.05]; ER− HR = 0.82 [0.74–0.91]). Duration of breastfeeding was associated with a trend toward higher risk of ER+ breast cancer (*P*_trend_ among ever breastfed = 0.02), but no significant associations were observed in categories of duration (e.g., ≥ 12 months vs. never, HR = 1.04 [0.97–1.11]). The associations between breastfeeding and ER− breast cancer risk were similar regardless of breastfeeding duration (e.g., ≥ 12 months vs. never, HR = 0.80 [0.70–0.92]; < 6 months vs. never, HR = 0.84 [0.75–0.95]; *P*_trend_ among ever breastfed = 0.35). Patterns were similar for ER+/PR+ and ER−/PR− breast cancer (Additional file 1: Table S1). Statistical adjustment for years between menarche and age at first birth and years between age at last birth and minimum of current age and age at menopause did not impact the results (data not shown).

We next considered the joint association of parity and breastfeeding and breast cancer risk by molecular phenotypes (Table 3). For luminal A breast cancer, parity of 3 or higher, relative to nulliparity, was associated with significantly lower risk only among women who breastfed (≥ 3 children, ever breastfed: HR = 0.84 [0.71–0.99]; never breastfed: HR = 0.93 [0.77–1.12]). In contrast, for luminal B breast cancer, the association between parity and breast cancer risk was similar regardless of breastfeeding history (e.g., ≥ 3 children, ever breastfed: HR = 0.78 [0.62–0.98]; never breastfed: HR = 0.76 [0.58–1.00]). We observed the suggestion of increased risk of basal-like tumors among parous women who never breastfed (e.g., ≥ 3 children, never breastfed: HR = 1.43 [0.92–2.23]); this increased risk was not observed among those who ever breastfed (HR = 1.05 [0.70–1.57]). Risk of HER2-enriched disease was higher in parous, as compared to nulliparous, women, regardless of breastfeeding (parous/never breastfed, HR = 2.09 [1.02–4.30]; parous/ever breastfed, HR = 1.90 [0.95–3.80]).

**Table 3:** Multivariable-adjusted^a^ hazard ratios (HRs) and 95% confidence intervals for molecular breast cancer subtypes in relation to parity and breastfeeding: Nurses’ Health Study (1976–2012) and Nurses’ Health Study II (1989–2013)

	Luminal A			Luminal B			HER2-enriched			Basal-like			*P*_het_ by subtype
	Cases	PY	HR (95%CI)	Cases	PY	HR (95%CI)	Cases	PY	HR (95%CI)	Cases	PY	HR (95%CI)	
Parity/breastfeeding													
Nulliparous	199	743,688	1 (ref.)	109	743,762	1 (ref.)	9	743,854	1 (ref.)	34	743,835	1 (ref.)	
Parous women, never breastfed	613	1,361,275	1.00 (0.85–1.18)	269	1,361,572	0.82 (0.65–1.04)	62	1,361,777	2.09 (1.02–4.30)	121	1,361,712	1.34 (0.90–2.00)	0.04^b^
1 child	89	210,499	1.07 (0.83–1.38)	48	210,527	1.11 (0.78–1.56)	13	210,562	3.35 (1.41–7.93)	14	210,562	1.08 (0.58–2.03)	0.03^c^
2 children	228	517,884	1.05 (0.87–1.28)	92	518,005	0.78 (0.58–1.03)	29	518,065	2.81 (1.31–6.05)	45	518,050	1.40 (0.89–2.22)	
≥ 3 children	296	632,891	0.93 (0.77–1.12)	129	633,040	0.76 (0.58–1.00)	20	633,150	1.29 (0.57–2.90)	62	633,100	1.43 (0.92–2.23)	
Parous women, ever breastfed	1091	3,187,450	0.87 (0.74–1.01)	556	3,187,935	0.80 (0.65–0.99)	115	3,188,338	1.90 (0.95–3.80)	186	3,188,280	0.96 (0.66–1.40)	
1 child	92	314,469	0.98 (0.76–1.26)	51	314,504	0.97 (0.70–1.36)	9	314,536	1.94 (0.77–4.92)	16	314,532	0.99 (0.54–1.80)	
2 children	340	1,168,513	0.87 (0.73–1.03)	174	1,168,664	0.77 (0.61–0.99)	26	1,168,794	1.43 (0.66–3.09)	55	1,168,780	0.84 (0.54–1.30)	
≥ 3 children	659	1,704,468	0.84 (0.71–0.99)	331	1,704,767	0.78 (0.62–0.98)	80	1,705,008	2.11 (1.03–4.29)	115	1,704,968	1.05 (0.70–1.57)	

^a^Adjusted for age, height (< 1.60, 1.60 to < 1.65, 1.65 to < 1.70, 1.70 to < 1.75, 1.75+ meters), BMI at age 18 (< 20, 20–21.9, 22–23.9, 24–26.9, 27+ kg/m^2^), weight change since age 18 (continuous, kg), age at menarche (< 12, 12, 13, 14, 15+ years), menopausal status (premenopausal, postmenopausal or unknown), age at natural menopause (continuous), HT use (never, past, current E only, current E + P, current other), alcohol consumption (non-drinker, < 5, 5–10, 10–15, 15+ g/day), total physical activity (< 3, 3–9, 9–18, 18–27, 27+ MET-h/day), family history of breast cancer (yes/no), history of benign breast disease (yes/no), age at first birth (continuous, years)

^b^*P*_het_ for nulliparous and parous ever vs. never breastfed

^c^*P*_het_ for nulliparous and number of children by ever vs. never breastfed

Overall, results were similar in the individual cohorts (NHS and NHSII). We observed significant heterogeneity between cohorts for ER+ disease (*p* = 0.02) in analyses considering the cross-classification of parity and breastfeeding. For ER+ disease, parity ≥ 3 among women who never breastfed was more strongly associated with breast cancer risk in the NHS (HR = 0.75 [0.67–0.84]) than in the NHSII (HR = 0.85 [0.67–1.08]); associations were similar in other categories (e.g., ≥3 children ever breastfed, NHS HR = 0.76 [0.69–0.85]; NHSII HR = 0.79 [0.69–0.89]).

Finally, we evaluated parity, breastfeeding, and breast cancer risk in strata of time between last pregnancy and diagnosis (< 10 vs. ≥ 10 years). Inverse associations between parity and ER+ disease and breastfeeding and ER− disease were observed only in women diagnosed 10+ years after last pregnancy (Additional file 1: Table S2). No associations were observed among participants diagnosed within 10 years of last pregnancy.

## Discussion

This large, prospective study provides additional evidence of lower risk of ER− breast cancer among women who have breastfed and, to our knowledge, is the first prospective investigation on the joint effect of parity and breastfeeding on breast cancer risk by molecular phenotype. While breastfeeding was inversely associated with ER− breast cancer, no association was observed for the ER+ subtype. In contrast, parity was associated with lower risk of ER+ breast cancer, but not associated with ER− disease. In analyses by molecular phenotype, higher parity was inversely associated with the luminal subtypes. Parity was associated with higher risk of basal-like disease among women who did not breastfeed and HER2-enriched breast cancer risk regardless of breastfeeding history. These results provide confirmatory evidence of heterogeneity in the associations between parity and breastfeeding and breast cancer subtypes and support a risk-reducing role of breastfeeding for ER- breast cancer.

The breast undergoes proliferation and differentiation during pregnancy, in preparation for lactation. Terminal differentiation of the terminal ductal lobular unit (TDLU) occurs in the final trimester; this is hypothesized to be an important mechanism linking full-term pregnancy to long-term reduced risk of breast cancer, shielding the breast tissue from carcinogenic transformation [26]. Recent research has identified parity-related gene expression signatures in normal breast tissue [27–29], providing further evidence for the long-term impact of pregnancy on the breast. A “parity signature” including differential expression in inflammatory and immune-related pathways may be retained in ER+, but not ER−, tumors [27] supporting a differential role for parity in the etiology of ER+ vs. ER− breast cancer. However, data are limited and the parity-related gene signatures are not entirely consistent across studies.

Direct data describing underlying physiologic mechanisms between breastfeeding and breast cancer subtypes are limited [30]. Post-pregnancy, and at cessation of lactation, the breast undergoes involution. Involution has features of wound healing [31, 32], which has established parallels to tumorigenic microenvironments, including involvement of cytokines, growth factors, and matrix metalloproteases (MMPs) (reviewed in [33]). Breastfeeding delays the process of involution, and it has been hypothesized that the more gradual involution associated with lactation (i.e., from exclusive breastfeeding through weaning) may reduce longer-term breast cancer risk. Further, the postpartum hormonal milieu differs in breastfeeding vs. non-breastfeeding women (e.g., breastfeeding is associated with lower estradiol and higher prolactin levels for up to 12 weeks postpartum and has been associated with a slower decline in postpartum osteoprotegerin levels) [34, 35]. This postpartum hormonal milieu may differentially impact risk in the context of involution in the immediate postpartum vs. after breastfeeding and may differentially impact development of ER+ vs. ER− disease. Finally, murine models show a lower percentage of ER+ luminal cells in the mammary glands of parous, relative to virgin, mice with no change in the number of basal-like progenitor cells, though these basal-like progenitor cells demonstrated characteristics suggesting reduced carcinogenic potential in parous mice [36]. Nonetheless, involution proximate to delivery (i.e., in the absence of breastfeeding) may potentiate basal-like progenitors toward a carcinogenic pathway.

While some prior research is suggestive of a positive association between parity and ER− breast cancer, data to date are not consistent (reviewed in [37]). The AMBER consortium is the largest study to date [11]. Parity was associated with 33% increased risk of ER− disease (*n* = 1252; parous vs. nulliparous, RR = 1.33 [1.11–1.59]). Higher risk was observed with higher parity among women who did not breastfeed (e.g., ≥ 4 vs. 1 birth, RR = 1.68 [1.15–2.44]) but not among women reporting ever breastfeeding (RR = 1.33 [0.91–1.95]). Ever parous, or number of children, were not significantly associated with ER− breast cancer in the current study, regardless of breastfeeding. Importantly, the AMBER consortium includes women of African-American descent, whereas the NHS/NHSII study population is predominantly non-Hispanic white. ER−/PR− (and triple negative) breast cancer is more common among African American women, as are higher parity [38] and lower rates of breastfeeding [39]. Consistent with most prior prospective studies, ever breastfeeding was significantly associated with lower risk of ER− disease, and not associated with ER+ disease, in the current study. These results are in line with a recent meta-analysis of prospective studies (ever vs. never, summary RRs: ER−/PR−, 0.84 [0.72–0.97]; ER+/PR+, 1.00 [0.90–1.10]) [10]. In analyses evaluating duration of breastfeeding, similar associations were observed between breastfeeding and ER− breast cancer regardless of duration, suggesting even shorter-term breastfeeding (cumulative < 6 months) may decrease ER− breast cancer risk. In contrast, a trend toward higher risk was observed between breastfeeding duration and ER+ breast cancer, though no significant associations were observed in any of the categories of duration. To our knowledge, this has not previously been reported. Previous prospective studies have not observed significant associations between breastfeeding duration and breast cancer risk by subtype [2, 12, 14–16]. Additional studies with detailed data on breastfeeding duration are warranted.

Few prior studies have examined the joint effects of parity and breastfeeding and breast cancer risk by molecular phenotype [5, 7, 9, 18]. Millikan et al., in the Carolina Breast Cancer Study [5], observed higher risk of basal-like breast cancer among women with high parity in the absence of breastfeeding (parity 3+, never breastfed: OR = 1.9 [1.1–3.3]); breastfeeding attenuated the increased risk (OR = 1.3 [0.7–2.3]). A case-only investigation reported similar associations [9]. In our prior analysis by molecular phenotype in the NHS/NHSII, we observed a positive association between parity and HER2-enriched disease, but no association with the other subtypes [13]. Breastfeeding was associated with a reduced risk of basal-like breast cancer (e.g., 7+ months vs. never, among parous women, HR = 0.65 [0.49–0.87]); however, the joint effects of parity and breastfeeding were not considered. In the current investigation, consistent with prior studies, parity was suggestively positively associated with basal-like breast cancer, but only among women with high parity and who never breastfed. The positive association of parity with HER2 disease did not differ by breastfeeding.

We provide the largest prospective study to date on parity, breastfeeding, and breast cancer by subtype, and add to the limited literature on these associations by molecular phenotype. While we expect that parity was accurately reported, there may be misclassification of breastfeeding duration. We would expect any misclassification to be nondifferential. Tumor tissue to define molecular phenotype was available for 33% of the cases, resulting in small case numbers in some subgroups.

## Conclusion

Our results support a role for breastfeeding in reducing risk of hormone receptor-negative breast cancer, adding to the established benefits of breastfeeding. While further research is needed to describe the mechanistic pathway resulting in this decreased risk, our investigation provides additional compelling evidence that breastfeeding is a modifiable risk factor for the breast cancer subtypes with the fewest targeted therapies and the least favorable outcomes.

## Additional file

Additional file 1: **Table S1.** Multivariate-adjusted^a^ hazard ratios (HRs) and 95% confidence intervals for breast cancer by ER/PR status in relation to parity and lactation: Nurses’ Health Study (1976–2012) and Nurses’ Health Study II (1989–2013). **Table S2.** Multivariate-adjusted^a^ hazard ratios (HRs) and 95% confidence intervals for breast cancer by ER status in relation to parity and lactation, stratified by time since last birth: Nurses’ Health Study (1976–2012) and Nurses’ Health Study II (1989–2013) (DOCX 30 kb)

## Abbreviations

AMBER: African American Breast Cancer Epidemiology and Risk (AMBER) Consortium
BMI: Body mass index
CI: Confidence interval
CK: Cytokeratin
EGFR: Epidermal growth factor receptor
ER: Estrogen receptor
HER2: Human epidermal growth factor receptor 2
HR: Hazard ratio
HT: Hormone therapy
MMP: Matrix metalloproteases
NHS: Nurses’ Health Study
OC: Oral contraceptive
PR: Progesterone receptor
TDLU: Terminal ductal lobular unit
TMA: Tissue microarray
