# Supplementary material for: Parity, breastfeeding, and breast cancer risk by hormone receptor status and molecular phenotype: results from the Nurses’ Health Studies
Source: Breast Cancer Res. 2019 Mar 12;21:40. doi: 10.1186/s13058-019-1119-y (PMC6416887; doi:10.1186/s13058-019-1119-y)
Supplement: Supplementary file 1 — Table S1. Multivariate-adjusteda hazard ratios (HRs) and 95% confidence intervals for breast cancer by ER/PR status in relation to parity and lactation: Nurses’ Health Study (1976–2012) and Nurses’ Health Study II (1989–2013). Table S2. Multivariate-adjusteda hazard ratios (HRs) and 95% confidence intervals for breast cancer by ER status in relation to parity and lactation, stratified by time since last birth: Nurses’ Health Study (1976–2012) and Nurses’ Health Study II (1989–2013) (DOCX 30 kb) [file 13058_2019_1119_MOESM1_ESM.docx]

| **Supplemental Table 1. Multivariable-adjusted^a^ hazard ratios (HRs) and 95% confidence intervals for breast cancer by ER/PR status in relation to parity and lactation: Nurses’ Health Study (1976-2012) and Nurses’ Health Study II (1989-2013)** | | | | | | | | | | | | |
| --- | --- | --- | --- | --- | --- | --- | --- | --- | --- | --- | --- | --- |
|  | **ER^+^/PR^+^** | | |  | **ER+/PR^-^** | | |  | **ER^-^/PR^-^** | | | **P_het_** |
|  | **Cases** | **PY** | **HR (95% CI)** |  | **Cases** | **PY** | **HR (95% CI)** |  | **Cases** | **PY** | **HR (95% CI)** | **by subtype** |
| **Parous** |  |  |  |  |  |  |  |  |  |  |  | 0.02 |
| Nulliparous | 811 | 743132 | 1 (ref.) |  | 139 | 743730 | 1 (ref.) |  | 171 | 743711 | 1 (ref.) |  |
| Parous | 5864 | 4544851 | 0.82 (0.76-0.89) |  | 1172 | 4549181 | 0.85 (0.71-1.02) |  | 1539 | 4548875 | 1.03 (0.88-1.22) |  |
|  |  |  |  |  |  |  |  |  |  |  |  |  |
| **Breastfeeding** |  |  |  |  |  |  |  |  |  |  |  |  |
| Never breastfed (Parous + nulliparous) | 2618 | 2103271 | 1 (ref.) |  | 547 | 2105192 | 1 (ref.) |  | 704 | 2105050 | 1 (ref.) | 0.01 |
| Ever breastfed | 4057 | 3184712 | 1.01 (0.95-1.07) |  | 764 | 3187719 | 0.91 (0.81-1.03) |  | 1006 | 3187536 | 0.85 (0.77-0.95) |  |
| ≤6 months | 1737 | 1257338 | 0.97 (0.91-1.04) |  | 369 | 1258566 | 0.91 (0.79-1.04) |  | 454 | 1258538 | 0.87 (0.76-0.98) |  |
| 7-11 months | 681 | 535545 | 1.06 (0.97-1.16) |  | 124 | 536052 | 0.93 (0.76-1.14) |  | 156 | 536016 | 0.81 (0.68-0.97) |  |
| ≥12 months | 1639 | 1391828 | 1.04 (0.97-1.12) |  | 271 | 1393102 | 0.91 (0.77-1.07) |  | 396 | 1392983 | 0.85 (0.75-0.98) |  |
|  |  |  | *P*_trend_ = 0.18 |  |  |  | *P*_trend_ = 0.70 |  |  |  | *P*_trend_ = 0.17 | 0.17 |
| **Parity/breastfeeding** |  |  |  |  |  |  |  |  |  |  |  |  |
| Nulliparous | 811 | 743132 | 1 (ref.) |  | 139 | 743730 | 1 (ref.) |  | 171 | 743711 | 1 (ref.) |  |
| Never breastfed | 1807 | 1360139 | 0.82 (0.75-0.89) |  | 408 | 1361462 | 0.91 (0.74-1.11) |  | 533 | 1361339 | 1.16 (0.97-1.38) | 0.003^b^ |
| 1 child | 268 | 210314 | 0.88 (0.76-1.01) |  | 56 | 210531 | 0.96 (0.70-1.32) |  | 74 | 210498 | 1.14 (0.86-1.50) | 0.02^c^ |
| 2 children | 703 | 517436 | 0.86 (0.77-0.95) |  | 140 | 517965 | 0.90 (0.71-1.14) |  | 215 | 517894 | 1.27 (1.03-1.56) |  |
| ≥3 children | 836 | 632389 | 0.75 (0.67-0.83) |  | 212 | 632967 | 0.88 (0.70-1.10) |  | 244 | 632947 | 1.08 (0.88-1.33) |  |
| Ever breastfed | 4057 | 3184712 | 0.82 (0.76-0.89) |  | 764 | 3187719 | 0.83 (0.69-1.00) |  | 1006 | 3187536 | 0.99 (0.84-1.17) |  |
| 1 child | 367 | 314221 | 0.94 (0.83-1.07) |  | 60 | 314482 | 0.91 (0.67-1.23) |  | 86 | 314469 | 1.04 (0.80-1.35) |  |
| 2 children | 1396 | 1167560 | 0.84 (0.77-0.92) |  | 252 | 1168590 | 0.88 (0.71-1.09) |  | 320 | 1168535 | 0.93 (0.77-1.12) |  |
| ≥3 children | 2294 | 1702932 | 0.78 (0.72-0.85) |  | 452 | 1704647 | 0.78 (0.64-0.95) |  | 600 | 1704532 | 1.01 (0.85-1.21) |  |
|  |  |  |  |  |  |  |  |  |  |  |  |  |
| **Among parous women** |  |  |  |  |  |  |  |  |  |  |  |  |
| **Parity^d^** |  |  |  |  |  |  |  |  |  |  |  |  |
| 1 child | 635 | 524535 | 1 (ref.) |  | 116 | 525012 | 1 (ref.) |  | 160 | 524967 | 1 (ref.) |  |
| 2 children | 2099 | 1684995 | 1.00 (0.91-1.10) |  | 392 | 1686555 | 1.06 (0.86-1.32) |  | 535 | 1686428 | 1.05 (0.87-1.26) |  |
| 3 children | 1712 | 1261187 | 1.01 (0.92-1.12) |  | 341 | 1262445 | 1.08 (0.86-1.35) |  | 429 | 1262376 | 1.06 (0.87-1.29) |  |
| ≥4 children | 1418 | 1074134 | 0.88 (0.80-0.98) |  | 323 | 1075169 | 1.00 (0.78-1.27) |  | 415 | 1075103 | 1.13 (0.92-1.38) |  |
|  |  |  | *P*_trend_ = 0.0003 |  |  |  | *P*_trend_ = 0.24 |  |  |  | *P*_trend_ = 0.35 | 0.05 |
| **Breastfeeding^d^** |  |  |  |  |  |  |  |  |  |  |  |  |
| Never breastfed | 1807 | 1360139 | 1 (ref.) |  | 408 | 1361462 | 1 (ref.) |  | 533 | 1361339 | 1 (ref.) | 0.005 |
| Ever breastfed | 4057 | 3184712 | 1.02 (0.96-1.08) |  | 764 | 3187719 | 0.91 (0.80-1.03) |  | 1006 | 3187536 | 0.84 (0.75-0.94) |  |
| ≤6 months | 1737 | 1257338 | 0.98 (0.91-1.04) |  | 369 | 1258566 | 0.91 (0.79-1.05) |  | 454 | 1258538 | 0.86 (0.76-0.98) |  |
| 7-11 months | 681 | 535545 | 1.05 (0.96-1.15) |  | 124 | 536052 | 0.92 (0.75-1.13) |  | 156 | 536016 | 0.80 (0.67-0.96) |  |
| ≥12 months | 1639 | 1391828 | 1.06 (0.98-1.14) |  | 271 | 1393102 | 0.90 (0.76-1.06) |  | 396 | 1392983 | 0.82 (0.71-0.95) |  |
|  |  |  | *P*_trend_, incl never =0.04(+) |  |  |  | *P*_trend_, incl never = 0.71 |  |  |  | *P*_trend_, incl never = 0.07 | 0.03 |
|  |  |  | *P*_trend_, among ever=0.03 |  |  |  | *P*_trend_, among ever=0.78 |  |  |  | *P*_trend_, among ever=0.51 |  |

^a^Adjusted for age, height (<1.60, 1.60-<1.65, 1.65-<1.70, 1.70-<1.75, 1.75+ meters), BMI at age 18 (<20, 20 - 21.9, 22 - 23.9, 24 - 26.9, 27+ kg/m^2^), weight change since age 18 (continuous, kg), age at menarche (<12, 12, 13, 14, 15+ years), menopausal status (premenopausal, postmenopausal or unknown), age at natural menopause (continuous), HT use (never, past, current E only, current E+P, current other), alcohol consumption (non-drinker, <5, 5-10, 10-15, 15+ g/day), total physical activity (<3, 3-9, 9-18, 18-27, 27+ MET-hr/day), family history of breast cancer (yes/no), history of benign breast disease (yes/no), age at first birth (continuous, years); ^b^P_het_ for nulliparous and parous ever vs. never breastfed; ^c^P_het_ for nulliparous and number of children by ever vs. never breastfed. ^d^Among parous women only, additionally adjusted for age at first birth (continuous, years), and parity and breastfeeding mutually adjusted.

| **Supplemental Table 2. Multivariable-adjusted^a^ hazard ratios (HRs) and 95% confidence intervals for breast cancer by ER status in relation to parity and lactation, stratified by time since last birth: Nurses’ Health Study (1976-2012) and Nurses’ Health Study II (1989-2013)** | | | | | | | | | | | | | |
| --- | --- | --- | --- | --- | --- | --- | --- | --- | --- | --- | --- | --- | --- |
|  | **ER^+^** | | | | | |  | **ER^-^** | | | | | |
|  | **< 10 years** | | | **>= 10 years** | | |  | **< 10 years** | | | **>= 10 years** | | |
|  | **Cases** | **PY** | **HR (95% CI)** | **Cases** | **PY** | **HR (95% CI)** |  | **Cases** | **PY** | **HR (95% CI)** | **Cases** | **PY** | **HR (95% CI)** |
| **Among parous women** | | | |  |  |  |  |  |  |  |  |  |  |
| **Parity** |  |  |  |  |  |  |  |  |  |  |  |  |  |
| 1 child | 91 | 141469 | 1 (ref.) | 678 | 382943 | 1 (ref.) |  | 44 | 141515 | 1 (ref.) | 144 | 383425 | 1 (ref.) |
| 2 children | 237 | 366998 | 1.08 (0.81-1.44) | 2327 | 1317572 | 0.98 (0.88-1.08) |  | 81 | 367129 | 0.77 (0.50-1.19) | 531 | 1319231 | 1.09 (0.88-1.35) |
| 3 children | 195 | 256163 | 1.13 (0.82-1.58) | 1925 | 1004628 | 0.97 (0.87-1.07) |  | 45 | 256314 | 0.53 (0.31-0.91) | 457 | 1005997 | 1.19 (0.95-1.49) |
| ≥4 children | 184 | 199052 | 0.93 (0.63-1.35) | 1619 | 874730 | 0.88 (0.79-0.99) |  | 63 | 199162 | 0.83 (0.44-1.54) | 404 | 875897 | 1.14 (0.89-1.45) |
|  |  |  | *P*_trend_ = 0.34 |  |  | *P*_trend_ = 0.003 |  |  |  | *P*_trend_ = 0.89 |  |  | *P*_trend_ = 0.51 |
| **Breastfeeding** |  |  |  |  |  |  |  |  |  |  |  |  |  |
| Never breastfed | 166 | 207951 | 1 (ref.) | 2126 | 1151749 | 1 (ref.) |  | 62 | 208057 | 1 (ref.) | 564 | 1153200 | 1 (ref.) |
| Ever breastfed | 541 | 755731 | 1.02 (0.84-1.25) | 4423 | 2428124 | 0.96 (0.90-1.02) |  | 171 | 756063 | 0.86 (0.61-1.20) | 972 | 2431349 | 0.80 (0.71-0.91) |
| ≤6 months | 161 | 207828 | 1.01 (0.80-1.27) | 2016 | 1049058 | 0.93 (0.87-1.00) |  | 51 | 207932 | 0.84 (0.56-1.26) | 466 | 1050550 | 0.86 (0.74-0.99) |
| 7-11 months | 86 | 131749 | 0.98 (0.73-1.31) | 733 | 403679 | 1.00 (0.91-1.11) |  | 31 | 131801 | 0.97 (0.61-1.56) | 149 | 404197 | 0.70 (0.57-0.87) |
| ≥12 months | 294 | 416154 | 1.06 (0.84-1.34) | 1674 | 975387 | 0.99 (0.91-1.07) |  | 89 | 416330 | 0.82 (0.55-1.23) | 357 | 976602 | 0.76 (0.64-0.90) |
|  |  |  | *P*_trend_ = 0.63 |  |  | *P*_trend_ = 0.38 |  |  |  | *P*_trend_ = 0.13 |  |  | *P*_trend_ = 0.03 |
| ^a^Adjusted for age, height (<1.60, 1.60-<1.65, 1.65-<1.70, 1.70-<1.75, 1.75+ meters), BMI at age 18 (<20, 20 - 21.9, 22 - 23.9, 24 - 26.9, 27+ kg/m^2^), weight change since age 18 (continuous, kg), age at menarche (<12, 12, 13, 14, 15+ years), menopausal status (premenopausal, postmenopausal or unknown), age at natural menopause (continuous), HT use (never, past, current E only, current E+P, current other), alcohol consumption (non-drinker, <5, 5-10, 10-15, 15+ g/day), total physical activity (<3, 3-9, 9-18, 18-27, 27+ MET-hr/day), family history of breast cancer (yes/no), history of benign breast disease (yes/no), age at first birth (continuous, years). | | | | | | | | | | | | | |
